# Road traffic noise and registry based use of sleep medication

**DOI:** 10.1186/s12940-017-0330-5

**Published:** 2017-10-23

**Authors:** Jorunn Evandt, Bente Oftedal, Norun Hjertager Krog, Svetlana Skurtveit, Per Nafstad, Per E. Schwarze, Eva Skovlund, Danny Houthuijs, Gunn Marit Aasvang

**Affiliations:** 1 Division of Infection Control and Environmental Health, Department of Air Pollution and Noise, Norwegian Institute of Public Health, P.O. Box 4404 Nydalen, N-0403 Oslo, Norway; 2 Division of Mental and Physical Health, Department of Mental Disorders, Norwegian Institute of Public Health, P.O. Box 4404 Nydalen, N-0403 Oslo, Norway; 3 Norwegian Centre for Addiction Research (SERAF), University of Oslo, P.O. Box N-1039 Blindern, 0315 Oslo, Norway; 4 Department of Community Medicine and Global Health, University of Oslo, Faculty of Medicine, P.O. Box N-1130 Blindern, 0318 Oslo, Norway; 5 Division of Mental and Physical Health, Department of Noncommunicable Diseases, Norwegian Institute of Public Health, P.O. Box 4404 Nydalen, N-0403 Oslo, Norway; 6 Department of Public Health and Nursing, Norwegian University of Science and Technology, Faculty of Medicine and Health Sciences, P.O. Box 8905, N-7491 Trondheim, Norway; 7 The Dutch National Institute for Public Health and the Environment (RIVM), P.O. Box 1, 3720 BA Bilthoven, the Netherlands

**Keywords:** Traffic noise, Prescription registry, Sleep medication, Hypnotics, Sleep, Insomnia, Directed acyclic graph, Population-based study.

## Abstract

**Background:**

Road traffic noise has been associated with adverse health effects including sleep disturbances. Use of sleep medication as an indicator of sleeping problems has rarely been explored in studies of the effects of traffic noise. Furthermore, using registry data on sleep medications provides an opportunity to study the effects of noise on sleep where attribution of sleep problems to noise is not possible.

**Methods:**

We used questionnaire data from the population-based study Health and Environment in Oslo (HELMILO) (2009–10) (*n* = 13,019). Individual data on sleep medications was obtained from the Norwegian Prescription Database (NorPD). Noise levels (*L*_night_) were modeled for the most exposed façade of the building at each participant’s home address. Logistic regression models adjusted for potential confounders were used to analyze the association between traffic noise and sleep medication use both for one whole year and for the summer season. The results were reported as changes in the effect estimate per 5 decibel (dB) increase in noise level.

**Results:**

We observed no association between traffic noise and sleep medication use during one year [odds ratio (OR) = 1.00; 95% confidence interval (CI): 0.96, 1.04]. For sleep medication use in the summer season, there was a positive, however non-significant association (OR = 1.04; 95% CI: 0.99, 1.10). Among individuals sleeping with the bedroom window open, the association increased slightly and was borderline statistically significant (OR = 1.06; 95% CI: 1.00, 1.12).

**Conclusions:**

We found no evidence of an association between traffic noise and sleep medication use during one year. However, for the summer season, there was some suggestive evidence of an association. These findings indicate that season may play a role in the association between traffic noise and sleep, possibly because indoor traffic noise levels are likely to be higher during summer due to more frequent window opening. More studies are, however, necessary in order to confirm this.

**Electronic supplementary material:**

The online version of this article (10.1186/s12940-017-0330-5) contains supplementary material, which is available to authorized users.

## Background

Sleep disturbances are considered the main health burden in relation to environmental noise exposure. The World Health Organization (WHO) has estimated that noise-induced sleep disturbances lead to nearly 1 million disability adjusted life-years (DALYs) in Western Europe [1]. Furthermore, an increasing number of people will be exposed to noise levels above guideline values as traffic volumes and urbanization continue to grow [2].

A number of both experimental and epidemiological studies have demonstrated an impact of road traffic noise on sleep disturbances such as difficulties falling asleep, awakenings, sleep stage changes, and autonomic responses [3–10]. Furthermore, poor sleep has been hypothesized to be a mediator in the association between noise and adverse health effects such as diabetes, adiposity, and myocardial infarction [11–14] as well as mental health problems [15–17].

Although there are strong indications of an association between traffic noise and sleep disturbances, studies on the association between road traffic noise and sleep medication use are scarce. In previous studies of noise and sleep medication use, the medication use has either been reported subjectively [10, 18, 19] or been drawn from a registry recording individual purchase of medications [20, 21]. The registry based studies have, however, not exclusively studied sleep medication use, but have also included other types of medication such as antidepressants and anxiolytics.

We have previously studied the association between traffic noise and self-reported sleep disturbances and self-reported sleep medication use, and found that traffic noise was related to difficulties falling asleep and waking up too early [10]. However, no association with self-reported sleep medication use was found [10]. Applying registry based sleep medication use as an outcome rather than sleep disturbances and medication use based on self-reports provides an opportunity to study the effects of noise on sleep avoiding attribution to noise. Other methods for studying the relation between noise and sleep unaffected by attribution to noise include physiological measurements of sleep such as polysomnography and actigraphy. These methods are, however, often not suitable for large samples. When the data on sleep medications are obtained from a prescription registry, the outcome will be based on a physician’s evaluation of the patients’ need for sleep medications. Furthermore, it can represent a patient’s coping strategy, since they visit a physician in order to get help with their sleeping problems. Registry data on sleep medications is also assumed to be a more valid outcome than self-reports, because self-reports are prone to information bias because the respondents could e.g. misunderstand how the drug in question is classified [22].

Noise annoyance has been found to vary according to season and a higher prevalence has been reported during summer than winter [23, 24]. These findings may be due to peoples’ tendency to keep their bedroom windows open during the warmer season, thereby increasing the indoor noise levels from outdoor sources. Although these studies specifically address noise annoyance, it is not unlikely that seasonal variations may influence other effects of traffic noise such as sleep disturbances and sleep medication use. Furthermore, a study on noise and blood pressure reported a stronger association at temperatures above 15 °C [25]. The authors further indicated that this possibly could be explained by higher indoor traffic noise levels as many people sleep with the window open during warm periods. To our knowledge, no previous study has assessed if the association between traffic noise and sleep outcomes could differ according to season.

A stronger association between traffic noise and sleep disturbances has previously been reported among individuals with high levels of trait anxiety [6]. This finding may also be relevant for sleep medication use, as it indicates that people with high levels of mental distress are particularly vulnerable to nighttime traffic noise.

In the present study, we investigated the association between road traffic noise and dispensed sleep medications from a prescription registry. Furthermore, we assessed whether the association was different during the summer season, than during the whole year. We also investigated whether the association differed according to degree of mental distress.

## Methods

### Study population

We used data from the Health and Environment in Oslo (HELMILO) study, conducted in 2009–10. In this study, a questionnaire was received by 27,097 Oslo inhabitants born in the years 1924–25, 1940–41, 1955, 1960 and 1970. The response rate was 48% (*n* = 13,019). By using the unique personal identification number assigned to all Norwegian citizens, the questionnaire data was linked to the geographic coordinates of each participant’s home address. A total of 881 participants were excluded because they had lived at their current address for less than one year, had moved out of Oslo before returning the questionnaire, or had not been assigned a noise exposure level due to missing or uncertain information about geographic coordinates of the residential address. The Regional Committee for Medical Research Ethics in Norway approved the study and each participant provided written informed consent.

### Registry based sleep medication use

Information on registry based sleep medication use was obtained from the Norwegian Prescription Database (NorPD). NorPD contains individual data on all prescription drugs dispensed at Norwegian pharmacies since 2004. The personal identification number makes it possible to access information on dispensed prescription drugs at an individual level [26]. We extracted data on dispensed hypnotics, coded as N05C according to the Anatomical Therapeutic Chemical (ATC) classification system [27]. A participant who had filled at least one prescription of hypnotics during the year 2009 was defined as a user of sleep medications.

The oldest participants in HELMILO were 84–85 years old. Most individuals of this age use some kind of prescription drugs [28]. However, the NorPD does not include individual level data on medications distributed at institutions. We therefore excluded those among the oldest who had not filled a prescription on any kind of drug in the year 2009 (*n* = 72), assuming they lived in institutions.

### Noise exposure assessment

Nighttime road and rail traffic noise (*L*_night_, A-weighted nighttime equivalent noise level, 2300–0700 h) was modeled for the most exposed façade of the building and assigned to each participant’s home address using geographic coordinates. The noise levels were calculated by the City of Oslo according to the Nordic Prediction Method for Road Traffic and Railway Noise, respectively [29–32]. Geographic information system (GIS) methodology was applied in the software package CadnaA [33]. The grids for the noise calculations were 5 × 5 m and calculation height was 4 m above terrain. Within each grid, the noise level was interpolated at points along the façade with 3 m distance. Road traffic data included in the model (traffic counts, percentage heavy vehicles, speed limits, diurnal distribution) were obtained from the Norwegian Public Roads Administration and the City of Oslo. Other inputs to the model were digitalized terrain data in 3D including topography, soft vs. hard ground, location of buildings, and noise screens. For railway noise, input data included traffic frequency, signed speed, and train type obtained from the Norwegian state-owned company, Bane NOR, responsible for traffic management of railway property. Similar data for tram and subway traffic was obtained from Sporveien Oslo AS, a municipally owned public transport operator in Oslo.

The noise exposure data we used, had originally been calculated for the strategic noise mapping of Oslo, which is conducted every five years in accordance with the European Environmental Noise Directive [34]. We had access to detailed continuous noise data calculated for the strategic noise mappings for the years 2006 and 2011. The noise mapping for 2011 [35] was the one performed closest in time to the study period (2009–2010), and therefore most participants were assigned a noise level for 2011. However, in the time between the study period and 2011, some buildings and noise screens had either been erected or demolished, and the road network had been restructured in some areas. Thus, in some cases the traffic noise data assessed for 2006 were evaluated as more representative than the 2011 data. We therefore assigned the noise level calculated for 2006 to the dwellings where the noise level had either increased or decreased substantially [+/− 3 dB (dB)] following these local changes (2.2% of dwellings).

### Covariates

In the procedure of selecting covariates to the statistical models, we used a directed acyclic graph (DAG) [36, 37]. The DAG was developed using the web-based software DAGitty [38]. In order to decide which variables to be included in the DAG, we reviewed previous relevant research. Since we considered sleep medication use as an indicator for sleep disturbances, we included variables relevant for the association between traffic noise exposure and sleep disturbances. In Additional file 1: Figure S1, we have presented a simplified version of the DAG. The full version of the DAG can be accessed at www.dagitty.net/mAeotvC. The minimal sufficient adjustment set we used for the statistical analyses included the variables age, sex, population density, marital status, alcohol use, smoking status, physical activity, night shift work, and rail traffic noise. Age and sex were specified as compulsory variables for the final adjustment set. Further, it has been found that socially deprived individuals are exposed to higher noise levels than more socially advantaged individuals [39]. Thus, we added the socioeconomic factors educational level and household income to the final adjustment set.

Information on age and sex was obtained from the National Population Registry. We obtained information on socioeconomic status (SES) from Statistics Norway. Educational level was categorized as low (elementary school / no formal education), medium (upper secondary education) and high (higher education). Pre-tax household income per year was categorized as Norwegian kroner (NOK) < 500,000, NOK 500,000-NOK < 1000,000, and NOK ≥ 1000,000. The area variable, population density, obtained from the City of Oslo, included the number of dwellings per km^2^ in the area of each participant’s home according to the following categories: < 1000 dwellings, 1000- < 1500 dwellings, 1500- < 2500 dwellings, and ≥2500 dwellings. For constructing the variable on marital status, we mainly used data from Statistics Norway, but added information on cohabitation obtained from the HELMILO questionnaire. Marital status was categorized as married/cohabiting, unmarried (never married), divorced/separated, and widow(er). The following lifestyle variables were obtained from the questionnaire: alcohol use the last year (never been drinking / not been drinking the last year, been drinking ≤3 times/month, 1–3 times/week, and 4–7 times/week), smoking status (current, previous, or never daily smoker), and physical activity [sedentary (mostly sedentary activities), moderately active (light physical activity at least two to four hours a week), and highly active (heavy physical activity at least four hours a week)]. Night shift work was categorized according to number of years the participants had worked regular night shifts (0 years, 1- < 5 years, and ≥5 years). Rail traffic noise, originally a continuous variable, was split into three categories: *L*_night_ < 35 dB, *L*_night_ 35 dB- < 45 dB, and *L*_night_ ≥ 45 dB.

### Statistical analyses

We used logistic regression for modeling the associations between road traffic noise and dispensed hypnotics. This association was investigated for sleep medication use for the year in total (2009) and for the summer season (June, July, and August). For each association, we ran two models. In Model 1, we adjusted for age and sex, and in Model 2, we adjusted for the variables identified in the DAG and the SES factors educational level and income. Observations with missing values on any of the variables in Model 2 were excluded.

Since one single prescription of sleep medications may be filled in conjunction with a major life event, and, thus, not reflect consistent use, we also performed the analyses where the outcome was having filled two prescriptions or more.

Because the noise levels were modeled at the most exposed façade of each participant’s home, we assumed that the noise exposure was more accurate among those having their bedroom facing a road. We obtained information on bedroom location from the questionnaire and we conducted additional analyses according to whether the participants’ bedrooms were facing a road or not.

The actual road traffic noise levels the participants were exposed to could vary according to window opening and closing habits. To address this issue, we performed separate analyses according to the participants’ reporting in the questionnaire whether they usually sleep with their bedroom window open or closed during summer. We performed these analyses both for the total study population as well as according to bedroom location in the building.

To assess whether mental distress could modify the association between traffic noise and sleep medication use, we stratified the sample according to high vs. low degree of mental distress. For measuring mental distress, we used the Hopkins Symptoms Checklist (HSCL) 10-item version, an abbreviated version of the HSCL-90-R [40]. The questionnaire consists of ten items that mainly taps into symptoms of anxiety and depression. A mean score of ≥1.85 on the HSCL-10 was considered a high degree of mental distress [41]. We performed this stratified analysis both for sleep medication use during the total year and for the summer season.

Since sleep medications were considered an indicator of sleep disturbances, we calculated the proportion of individuals having filled a prescription of sleep medications during 2009 that also reported to have sleep disturbances. Self-reported sleep disturbances were reported in the HELMILO questionnaire and we used the three following items: difficulties falling asleep, awakenings during the night, and waking up too early at a frequency of at least 3–5 times per week. The same frequency was used in a previous study were these sleep problems were used as outcomes [10].

Indications that noise exposure may affect sleep differently in men and women have been reported [42]. Hence, it is possible that similar differences could be relevant for sleep medication use. We therefore tested the interaction between road traffic noise and sex on sleep medication use by means of the log-likelihood test and performed sex-stratified analyses.

All results are reported as changes in the effect estimate per 5 dB increase in noise level. A 5% level of significance was used for all statistical analyses, except for the interaction tests for which we used a 10% level. Statistical analyses were carried out in STATA version 14 (StataCorp, College Station, Texas, USA). We visualized the associations between road traffic noise and hypnotics use by applying a smooth function of the associations with non-parametric regression spline as smoother. Such models are named generalized additive models (GAM). The function gam in library mgcv in the R statistical software version 3.3.2 (The R Project for Statistical Computing, Vienna, Austria) was used to estimate the splines with 95% confidence limits.

## Results

In the total study population, 14.1% (*n* = 1698) had filled at least one prescription of hypnotics in the course of a whole year. The corresponding proportion for the summer season was 6.7% (*n* = 808). In Table 1, we have presented the distribution of number of prescriptions, both for the year 2009 in total and for the summer season.

**Table 1:** Number (percentage) of filled prescriptions of hypnotics for the year in total and for the summer season

No. prescriptions	The year in total	Summer season
1	665 (5.5)	600 (5.0)
2	337 (2.8)	106 (0.9)
3	217 (1.8)	49 (0.4)
4	195 (1.6)	13 (0.1)
≥5	284 (2.4)	40 (0.3)

The modeled road traffic noise levels ranged from *L*_night_ 7.6 dB to *L*_night_ 70.8 dB with a mean of *L*_night_ 47.2 dB (SD = ± 8.0). Table 2 shows a detailed description of the characteristics of the total study population and by three categories of road traffic noise exposure. The most pronounced differences across noise exposure categories were seen for the covariates marital status, household income, smoking status, physical activity, population density, and rail traffic noise.

**Table 2:** Characteristics of the study population by nighttime road traffic noise (*L*_night_) exposure

		Nighttime road traffic noise (*L*_night_) *n* (%)			
Characteristic (no. missing)		< 45 dB 4285 (35.5)	45- < 55 dB 5882 (48.7)	≥ 55 dB 1899 (15.7)	Total 12,066 (100)
Age (0)	39 years	793 (18.5)	1196 (20.3)	457 (24.1)	2446 (20.3)
	49 years	1035 (24.2)	1257 (21.4)	393 (20.7)	2685 (22.3)
	54 years	942 (22.0)	1215 (20.7)	354 (18.6)	2511 (20.8)
	68–69 years	1123 (26.2)	1562 (26.6)	504 (26.5)	3189 (26.4)
	84–85 years	392 (9.1)	652 (11.1)	191 (10.1)	1235 (10.2)
Sex (0)	Women	2320 (54.1)	3156 (53.7)	1040 (54.8)	6516 (54.0)
	Men	1965 (45.9)	2726 (46.3)	859 (45.2)	5550 (46.0)
Marital status (1)	Married/cohabiting	3238 (75.6)	3946 (67.1)	1058 (55.7)	8242 (68.3)
	Unmarried	352 (8.2)	803 (13.7)	410 (21.6)	1565 (13.0)
	Divorced/separated	437 (10.2)	728 (12.4)	281 (14.8)	1446 (12.0)
	Widow(er)	258 (6.0)	404 (6.9)	150 (7.9)	812 (6.7)
Educational level (86)	Low	523 (12.3)	927 (15.9)	319 (16.9)	1769 (14.8)
	Medium	1501 (35.3)	2127 (36.4)	671 (35.6)	4299 (35.9)
	High	2230 (52.4)	2786 (47.7)	896 (47.5)	5912 (49.3)
Household income (13)	NOK < 500 k	961 (22.4)	1794 (30.5)	725 (38.3)	3480 (28.9)
	NOK 500 k- < 1000 k	1613 (37.7)	2359 (40.1)	742 (39.2)	4714 (39.1)
	NOK ≥ 1000 k	1708 (39.9)	1724 (29.3)	427 (22.5)	3859 (32.0)
Population density (0)	< 1000 dwellings/km^2^	1107 (25.8)	905 (15.4)	147 (7.7)	2159 (17.9)
	1000–1500 dwellings/km^2^	1053 (24.6)	1234 (21.0)	277 (14.6)	2564 (21.2)
	1500–2500 dwellings/km^2^	1584 (37.0)	1600 (27.2)	342 (18.0)	3526 (29.2)
	≥ 2500 dwellings/km^2^	541 (12.6)	2143 (36.4)	1133 (59.7)	3817 (31.6)
Alcohol use (77)	Never / not last year	316 (7.4)	452 (7.7)	178 (9.5)	946 (7.9)
	≤ 3 times / month	1264 (29.7)	1793 (30.6)	613 (32.6)	3670 (30.6)
	1–3 times / week	2130 (50.1)	2791 (47.7)	802 (42.6)	5723 (47.7)
	4–7 times / week	545 (12.8)	815 (13.9)	290 (15.4)	1650 (13.8)
Smoking status (112)	Current	582 (13.7)	945 (16.2)	354 (18.8)	1881 (15.7)
	Former	1565 (36.8)	2225 (38.2)	705 (37.4)	4495 (37.6)
	Never	2103 (49.5)	2651 (45.5)	824 (43.8)	5578 (46.7)
Physical activity (267)	Sedentary	366 (8.7)	638 (11.1)	234 (12.6)	1238 (10.5)
	Moderately active	2761 (65.7)	3779 (65.7)	1246 (67.3)	7786 (66.0)
	Highly active	1073 (25.5)	1331 (23.2)	371 (20.0)	2775 (23.5)
Night shift work (194)	0 years	3532 (83.7)	4787 (82.8)	1515 (80.9)	9834 (82.8)
	1- < 5 years	385 (9.1)	493 (8.5)	179 (9.6)	1057 (8.9)
	≥ 5 years	304 (7.2)	499 (8.6)	178 (9.5)	981 (8.3)
Rail traffic noise (0)	< 35 dB	3602 (84.1)	4492 (76.4)	1175 (61.9)	9269 (76.8)
	35 dB- < 45 dB	447 (10.4)	903 (15.4)	290 (15.3)	1640 (13.6)
	≥ 45 dB	236 (5.5)	487 (8.3)	434 (22.9)	1157 (9.6)
Sleep medication total year (0)	Yes	578 (13.5)	827 (14.1)	293 (15.4)	1698 (14.1)
	No	3707 (86.5)	5055 (85.9)	1606 (84.6)	10,368 (85.9)
Sleep medication summer (0)	Yes	259 (6.0)	382 (6.5)	167 (8.8)	808 (6.7)
	No	4026 (94.0)	5500 (93.5)	1732 (91.2)	11,258 (93.3)

*Abbreviations*: *dB* decibel, *NOK* Norwegian kroner

A total of 88.8% (*n* = 10,681) reported to sleep with their bedroom window kept open during summer and 33.0% (*n* = 3952) reported to have their bedroom facing a road. Among those having the bedroom facing a road, the noise levels ranged from *L*_night_ 19.1 dB to *L*_night_ 70.4 dB with a mean of *L*_night_ dB 49.7 (SD = ±7.3), which is higher than for the total study population. For sleep medication use during one year, there was a slightly higher proportion having filled a prescription on hypnotics (15.6%) in the group having the bedroom facing a road than in the total study population. Furthermore, of the total study population, 11.8% (*n* = 1337) was in the category of high degree of mental distress.

**Fig. 1:**
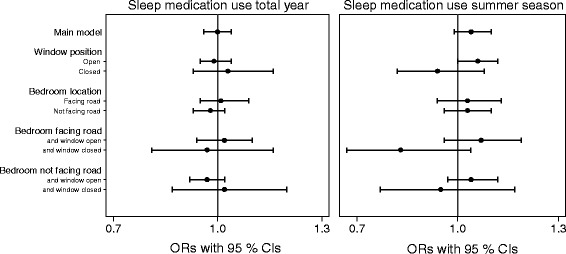
Associations between nighttime road traffic noise and sleep medication use by 5 dB increase in noise level. All models are adjusted for age, sex, educational level, household income, population density, marital status, alcohol use, smoking status, physical activity, night shift work, and rail traffic noise. The horizontal whiskers show ORs with 95% CIs

Fig. 1 shows the results from the regression analyses for sleep medication use during the total year and during the summer season, where all analyses are adjusted for potential confounders. The results from the analysis of sleep medication use during the total year indicated no statistically significant association with road traffic noise [odds ratio (OR) = 1.00; 95% confidence interval (CI): 0.96, 1.04]. Regarding sleep medication use during the summer season, we observed a borderline statistically significant association among those sleeping with the bedroom window open (OR = 1.06; 95% CI: 1.00, 1.12). There was a negative, but not statistically significant association among those keeping their bedroom window closed (OR = 0.94; 95% CI: 0.82, 1.08). Furthermore, when stratifying on window position within the group having the bedroom facing a road, we observed an even stronger negative effect estimate among those sleeping with the bedroom window closed (OR = 0.83; 95% CI: 0.67, 1.04). Among those sleeping with the bedroom window open, the effect estimate remained similar to the group keeping the window open within the total study population (OR = 1.07; 95% CI: 0.96, 1.19). In the analyses stratified on bedroom location, no difference in the effect estimate was shown. A detailed overview of the results is shown in Additional file 2: Table S1.

Of the total study population, there was 1033 (8.6%) participants who had filled two or more prescriptions on hypnotics during 2009. A total of 208 (1.7%) had filled two or more prescriptions during the summer season 2009. When using two or more prescriptions as the outcome for one year in total, the effect estimate remained similar, compared to the main analysis (OR = 0.99; 95% CI: 0.94, 1.04). For the summer season, we observed a reduction in the estimate by using two or more prescriptions as the outcome (OR = 0.98; 95% CI: 0.88, 1.08).

**Fig. 2:**
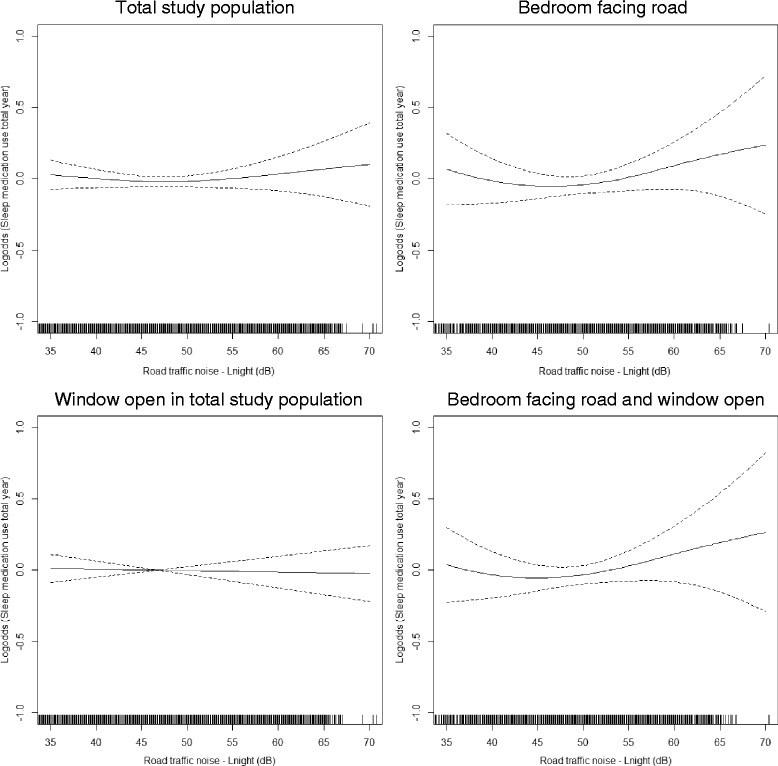
Splines with 95% confidence limits of the associations between nighttime road traffic noise shown from 35 dB and sleep medication use for the total year. The vertical lines on the x-axis indicate number of observations. General additive model adjusted for age, sex, educational level, household income, population density, marital status, alcohol use, smoking status, physical activity, night shift work, and rail traffic noise

**Fig. 3:**
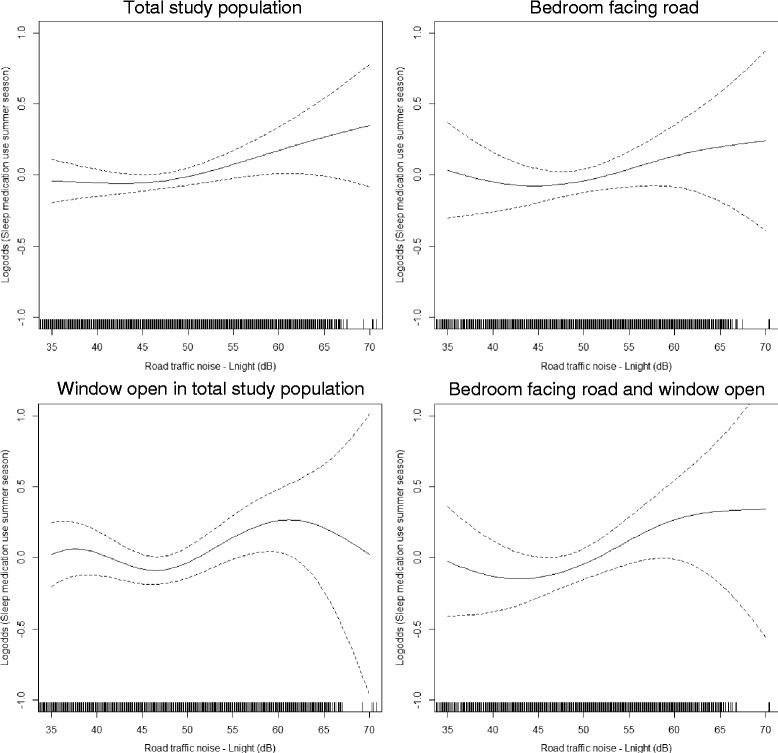
Splines with 95% confidence limits of the associations between nighttime road traffic noise shown from 35 dB and sleep medication use during summer season. The vertical lines on the x-axis indicate number of observations. General additive model adjusted for age, sex, educational level, household income, population density, marital status, alcohol use, smoking status, physical activity, night shift work, and rail traffic noise

Visual assessment of the association between noise and medication use during the total year indicated a slight exposure-response relationship from around *L*_night_ 50 dB among individuals with their bedroom facing a road (Fig. 2). For the summer season, an exposure-response relationship from around *L*_night_ 45 dB was indicated in the total study population (Fig. 3). Furthermore, among those also sleeping with the window open, the association seemed more unclear with an increase from around *L*_night_ 50 dB and, then, a decrease from around *L*_night_ 65 dB. In the group having their bedroom facing a road, the association increased slightly from around *L*_night_ 50 dB, but then leveled off around *L*_night_ 60 dB. A similar tendency was shown among those who both had the bedroom facing a road and kept the window open.

In the analyses stratified on high vs. low degree of mental distress, the effect estimates were similar for sleep medication use during the total year. For sleep medication use in the summer season, on the other hand, we observed a higher effect estimate among those with a high degree of mental distress (OR = 1.09; 95% CI: 0.98, 1.21) than among those with low mental distress (OR = 1.03; 95% CI: 0.96, 1.10). However, these results were not statistically significant and the confidence intervals of the two strata also overlapped.

In assessing the relation between sleep disturbances and sleep medication use, we found that 590 (46%) among the participants having difficulties falling asleep had filled a prescription of sleep medications in 2009. The corresponding proportions for those reporting awakenings during the night and to wake up too early were 537 (35.8%) and 437 (34.0%), respectively.

The interaction tests between traffic noise exposure and sex on sleep medication use were not statistically significant for either the analysis of sleep medication use during the total year (*p* = 0.42) or the summer season (*p* = 0.28). The results from the sex-stratified analyses are shown in Additional file 1: Table S1.

## Discussion

In the present study, we used registry data for investigating the association between nighttime road traffic noise and sleep medication use. This association was assessed both for sleep medication use for the total year and for the summer season. Our findings showed no association between traffic noise and sleep medication use during the total year. Medication use during the summer season was positively, but not statistically significantly related to traffic noise. According to degree of mental distress, there was no difference in the association for sleep medication use during the total year. Although we observed a difference in effect estimates for the summer season, there was no clear evidence of a stronger association among those with a high vs. low degree of mental distress.

A previous study on road traffic noise and registry based sleep medication use reported no statistically significant association [21], which is in accordance with our findings. This study did, however, include anxiolytics and antidepressants in addition to hypnotics in the outcome. Another study that included both hypnotics and anxiolytics reported an association with traffic noise, but only among individuals living in areas low in social deprivation [20]. Because we considered sleep medication use as an indicator of sleep problems, hypnotics were the only drug included as outcome in the present study. Although anxiolytics and antidepressants may be used as sleep medications, hypnotics are likely to reflect sleep disturbances more specifically since treatment of sleep disturbances is its main function. Furthermore, hypnotics are the most commonly prescribed drug to treat sleep problems in Norway [43]. Previous studies having applied self-reported sleep medication use reported no association with road traffic noise [10, 18, 19]. The focus of the present study is on road traffic noise, but sleep medication use has also been studied in relation to other modes of traffic noise. One study on rail traffic noise reported an effect on self-reported sleep medication use the last year [44]. Furthermore, aircraft noise in the late evening has been associated with self-reported use of non-prescribed sleep medications [45].

In the analyses using the outcome of two or more prescriptions, the effect estimate remained similar to the main analysis for one year in total. For the summer season, on the other hand, the effect estimate was reduced and there was no longer a positive association between noise and medication use. Only a very small proportion (< 2%) of the study population had filled two or more prescriptions during the three summer months, which may partly explain the reduction in the association.

In the present study, the association between noise and medication use was slightly more pronounced for the summer season than for the total year. This can be seen in context with a previous study that found that noise annoyance was more frequently reported in the summer season than during the winter season [24]. Further, we found that the association between noise and sleep medication use during the summer season was stronger among those sleeping with the bedroom window open, than for the total study population. In the Nordic countries, it is more common to keep the bedroom window open during summer than other parts of the year, which may explain why we did not see a similar association for the total year. Among those sleeping with the bedroom window closed we observed a negative association, indicating a protective effect. When stratifying on window opening and closing among those having the bedroom facing a road, this negative effect was even further strengthened. The explanation may be that the difference in indoor and outdoor nighttime road traffic noise is likely to be larger when the bedroom is facing a road compared to a shielded side. In contrast, a study by Babisch and co-workers [46] found that individuals keeping the bedroom window closed were more annoyed by noise than those keeping the window open. According to the authors’ interpretation, window closing served as an indicator of perceived annoyance rather than a modifier reducing annoyance. However, a similar effect of window position was not found for the association between road traffic noise and hypertension [46]. Nevertheless, by keeping the bedroom window closed, the indoor level from outdoor road traffic noise will be reduced considerably. The conflicting results regarding the effect of window opening and closing habits on noise and health associations may reflect the complexity of such possible coping mechanisms, and that the effect on, or of such strategies may differ depending on the health outcome under study.

The analyses stratified on bedroom location did not show any notable differences in the effect estimates. This is contrary to what we expected since noise exposure is commonly estimated for the most exposed façade of the building and the bedroom could be located at a shielded facade. Although sleep medication use is not completely comparable to self-reported sleep disturbances, we found higher estimates for the group with the bedroom facing a road in a study on noise and sleep disturbances [10]. Furthermore, it has been pointed out that bedroom location should be taken into account in order not to underestimate the true effect of noise on sleep [5, 47–49]. As far as we know, this is the first study on road traffic noise and registry based sleep medication use to include window opening behavior and bedroom location in the analyses.

We observed a higher effect estimate in the association between noise and sleep medication use among those with a high vs. low degree of mental distress. However, the confidence intervals overlapped, so the difference in the estimates may be due to random variation. In line with our findings, a previous study on traffic noise and psychotropic medication use, including sleep medications, reported no difference in the association according to level of anxiety score [21]. However, a stronger association between noise and sleep disturbances has been reported for individuals with high vs. low trait anxiety [6]. Although sleep medication use may be a proxy for sleep disturbances, this outcome may represent more severe sleep disorders [50]. Thus, our finding may be due to a less clear association between noise and sleep medication use than between noise and self-reported sleep disturbances.

Because of the well-established association between traffic noise and sleep disturbances, we expected to find an association between road traffic noise and sleep medication use. However, not all people suffering from sleeping problems may use prescribed sleep medication. In the present study, we found that less than half of the participants reporting any of the sleep problems difficulties falling asleep, awakenings during the night, or waking up too early had filled a prescription on sleep medications. Further, sleep medication use may represent, or at least include, more severe perceived sleep disturbances not strongly associated with noise. Moreover, the use of sleep medication can potentially affect how someone would respond to questionnaire items on sleep disturbances. In our previous paper on self-reported sleep disturbances and sleep medication use [10] we discussed the possibility that someone using sleep medications might respond to sleep well because of the effect of the medication. On the other hand, it is also possible that a sleep medication user will report poor sleep because of the need for medication in order to sleep well. Thus, the relationship between sleep medication use and self-reported sleep is not easy to interpret.

A strength of the present study entail using data from a large population-based study (HELMILO). Furthermore, the HELMILO questionnaire was specifically designed to examine health effects from environmental exposures. Information bias was prevented by using modeled noise exposure levels and registry data on sleep medications obtained independently both of each other, and other questionnaire data. The noise exposure was thoroughly assessed, using a detailed noise model. Furthermore, the study population included participants from both urban and suburban areas of the City of Oslo. This resulted in a broad range of noise exposure levels, which strengthened the possibility to detect associations. A common approach in studies on noise and health is to use a cut off level for the noise exposure in order to account for background noise. In the present study, we found it appropriate to use the full range of exposure levels in the analyses. Firstly, because the only noise source included in the noise model is road traffic noise. Thus, by increasing the lowest modeled noise levels to a level of background noise, misclassification of exposure would likely occur, the mean exposure level in the study population would increase, and the association between road traffic noise and sleep medication use could potentially be overestimated. Furthermore, a cut off would also mean that some of the variance in the exposure is lost and the accuracy of the analytic model will consequently be reduced.

In NorPD, the date of each dispensed drug is registered. This enabled us to study sleep medication use over specific periods of time, such as during one year and the summer season. This is often not possible in self-report questionnaires, where medication use is commonly reported for a fixed period of time such as the last year.

Since we had questionnaire information about whether the bedroom was facing a road or a shielded side of the building we were able to perform analyses according to location of bedroom. This could potentially reduce exposure misclassification since the noise exposure was assessed for the most exposed façade. Furthermore, we had access to a large set of potential confounders from the questionnaire, the City of Oslo, and Statistics Norway, including population density and variables on SES. To select confounders for the statistical model, we applied the DAG framework, a thorough procedure for confounder selection in order to minimize bias [51].

A limitation with the present study is that it is of cross-sectional design, and we therefore cannot ascertain that the noise exposure precedes hypnotics use. However, in our analytic sample we only included the individuals that had lived at their current home address for more than one year. Hence, the noise exposure is likely to have been stable for some time. The study had a response rate of 48%, which is not optimal regarding representativeness. Although the generalizability of the study may be affected by a low response rate, this is only the case if the relationship between exposure and outcome is different among the study participants and those who did not participate [52]. We have no reason to assume such differences. Further, we did not have information on sound insulation factors such as type of bedroom window, ventilation, and façade insulation, which affects the transmission of outdoor traffic noise to noise levels inside the bedroom. However, the sound insulating effect of these factors are highly minimized when the windows are kept open, which was the case for the majority of our study sample, as 89% reported to keep their bedroom window open during the night. Nevertheless, we cannot rule out the possibility that some exposure misclassification has occurred.

A limitation in using registry data on medications is that registries on prescription drugs commonly only include information on whether a drug has been dispensed. Hence, there is no information on whether the medication is actually being used. Still, we consider it likely that a person who obtains a prescription on hypnotics and fills it at a pharmacy experiences sleep disturbances and has the intention of using the medication.

In the analyses of sleep medication use during the summer season, the noise levels should optimally have reflected this time of the year, however no such data were available. Still, the noise metric that we used, *L*_night_, is commonly used in scientific studies and is currently used by WHO for providing guidelines for nighttime noise [53].

## Conclusions

The results of the present study suggest no association between nighttime road traffic noise and sleep medication use during one year. Furthermore, there was no indication that the association differed according to degree of mental distress. These main findings may indicate that sleep medication use possibly represent more severe sleep problems not strongly associated with road traffic noise.

The findings of a weak, but positive association for the summer season, but not for the total year may be explained by higher indoor noise levels during summer, as windows are kept open more often. This further indicates that season may play a role in the relation between traffic noise and sleep medication use. Thus, future studies should take seasonal variations into account. Furthermore, bedroom location as well as window opening and closing behavior are important variables to take into account in order to reduce exposure misclassification in future studies on long-term effects of noise on sleep.

## Additional file

Additional file 1: Figure S1. Simplified directed acyclic graph for the association between road traffic noise and sleep medication use. Some variables have been grouped for legibility. a Includes the variables age, sex, and having children ≤ 5 years b Includes the variables education and household income c Includes the variables smoking status, alcohol use, caffeine use, physical activity, and night shift work. (DOCX 64 kb)
Additional file 2: Table S1. ORs and 95 % CIs for the association between nighttime road traffic noise (Lnight) and sleep medication use by 5 dB increase in noise level. (DOCX 24 kb)

## Abbreviations

ATC: Anatomical Therapeutic Chemical
CI: Confidence interval
DAG: Directed acyclic graph
DALY: Disability adjusted life-year
dB: decibel
GAM: Generalized Addictive Model
GIS: Geographic Information System
HELMILO: The Health and Environment in Oslo Study
HSCL: Hopkins Symptoms Checklist
NOK: Norwegian kroner
NorPD: Norwegian Prescription Database
OR: Odds ratio
SES: Socioeconomic status
WHO: World Health Organization
