# Supplementary material for: Road traffic noise and registry based use of sleep medication
Source: Environ Health. 2017 Oct 23;16:110. doi: 10.1186/s12940-017-0330-5 (PMC5660445; doi:10.1186/s12940-017-0330-5)
Supplement: Additional file 1: Figure S1. — Simplified directed acyclic graph for the association between road traffic noise and sleep medication use. Some variables have been grouped for legibility. a Includes the variables age, sex, and having children ≤ 5 years b Includes the variables education and household income c Includes the variables smoking status, alcohol use, caffeine use, physical activity, and night shift work. (DOCX 64 kb) [file 12940_2017_330_MOESM1_ESM.docx]

**Additional file 1:**

Road Traffic Noise and Registry Based Use of Sleep Medication

**Neighborhood noise**

**Demography**^a^

**Noise annoyance**

**Population density**

**Rail traffic noise**

**Noise sensitivity**

**Lifestyle**^c^

**Sleep medication use**

**Chronic diseases**

**Socioeconomic status**^b^

**Road traffic noise**

**Figure S1**. Simplified directed acyclic graph for the association between road traffic noise and sleep medication use. Some variables have been grouped for legibility.

^a^Includes the variables age, sex, and having children ≤ 5 years

^b^Includes the variables education and household income

^c^Includes the variables smoking status, alcohol use, caffeine use, physical activity, and night shift work
