# Supplementary material for: Road traffic noise and registry based use of sleep medication
Source: Environ Health. 2017 Oct 23;16:110. doi: 10.1186/s12940-017-0330-5 (PMC5660445; doi:10.1186/s12940-017-0330-5)
Supplement: Additional file 2: Table S1. — ORs and 95 % CIs for the association between nighttime road traffic noise (Lnight) and sleep medication use by 5 dB increase in noise level. (DOCX 24 kb) [file 12940_2017_330_MOESM2_ESM.docx]

| **Table S1**. ORs and 95 % CIs for the association between nighttime road traffic noise (*L*_night_) and sleep medication use by 5 dB increase in noise level. | | | | | | |
| --- | --- | --- | --- | --- | --- | --- |
|  |  | **Total year** | |  | **Summer season** | |
|  | **n** | **Model 1^a^**  **OR (95 % CI)** | **Model 2^b^**  **OR (95 % CI)** |  | **Model 1^a^**  **OR (95 % CI)** | **Model 2^b^**  **OR (95 % CI)** |
| **Total study population** | 11,420 | 1.03 (1.00, 1.07) | 1.00 (0.96, 1.04) |  | 1.10 (1.04, 1.15) | 1.04 (0.99, 1.10) |
| **Bedroom window open** | 10,142 | 1.03 (0.99, 1.07) | 0.99 (0.95, 1.04) |  | 1.10 (1.05, 1.16) | 1.06 (1.00, 1.12) |
| **Bedroom window closed** | 1,244 | 1.08 (0.98, 1.19) | 1.03 (0.93, 1.16) |  | 1.03 (0.91, 1.16) | 0.94 (0.82, 1.08) |
| **Bedroom facing road** | 3,722 | 1.05 (0.98, 1.11) | 1.01 (0.95, 1.09) |  | 1.06 (0.97, 1.16) | 1.03 (0.94, 1.13) |
| **Bedroom not facing road** | 7,648 | 1.01 (0.97, 1.06) | 0.98 (0.93, 1.02) |  | 1.09 (1.03, 1.16) | 1.03 (0.96, 1.10) |
| **Bedroom facing road and window open** | 3,171 | 1.04 (0.97, 1.11) | 1.02 (0.94, 1.10) |  | 1.08 (0.98, 1.19) | 1.07 (0.96, 1.19) |
| **Bedroom facing road and window closed** | 539 | 1.05 (0.90, 1.23) | 0.97 (0.81, 1.16) |  | 0.92 (0.76, 1.11) | 0.83 (0.67, 1.04) |
| **Bedroom not facing road and window open** | 6,935 | 1.01 (0.96, 1.06) | 0.97 (0.92,1.02) |  | 1.10 (1.03, 1.18) | 1.04 (0.97, 1.12) |
| **Bedroom not facing road and window closed** | 693 | 1.08 (0.94, 1.23) | 1.02 (0.87, 1.20) |  | 1.06 (0.88, 1.27) | 0.95 (0.77, 1.17) |
| **High mental distress** | 1,260 | 0.99 (0.92, 1.07) | 0.99 (0.91, 1.08) |  | 1.11 (1.00, 1.22) | 1.09 (0.98, 1.21) |
| **Low mental distress** | 9,568 | 1.03 (0.99, 1.07) | 1.00 (0.95,1.05) |  | 1.07 (1.01, 1.14) | 1.03 (0.96, 1.10) |
| **Women** | 6,097 | 1.02 (0.98, 1.07) | 1.00 (0.95, 1.04) |  | 1.07 (1.01, 1.14) | 1.02 (0.96, 1.09) |
| **Men** | 5,323 | 1.06 (1.00, 1.12) | 1.00 (0.94, 1.07) |  | 1.15 (1.06, 1.25) | 1.07 (0.98, 1.18) |
| Abbreviations: dB, decibel; OR, odds ratio; CI, confidence interval. ^a^Model adjusted for age and sex. ^b^Model adjusted for age, sex, educational level, household income, population density, marital status, alcohol use, smoking status, physical activity, night shift work, and rail traffic noise. | | | | | | |
